# Chronic Stress: Impacts on Tumor Microenvironment and Implications for Anti-Cancer Treatments

**DOI:** 10.3389/fcell.2021.777018

**Published:** 2021-11-19

**Authors:** Wentao Tian, Yi Liu, Chenghui Cao, Yue Zeng, Yue Pan, Xiaohan Liu, Yurong Peng, Fang Wu

**Affiliations:** ^1^ Department of Oncology, The Second Xiangya Hospital, Central South University, Changsha, China; ^2^ Xiangya School of Medicine, Central South University, Changsha, China; ^3^ Xiangya School of Public Health, Central South University, Changsha, China; ^4^ Hunan Cancer Mega-Data Intelligent Application and Engineering Research Centre, Changsha, China; ^5^ Hunan Key Laboratory of Tumor Models and Individualized Medicine, The Second Xiangya Hospital, Central South University, Changsha, China; ^6^ Hunan Key Laboratory of Early Diagnosis and Precision Therapy in Lung Cancer, The Second Xiangya Hospital, Central South University, Changsha, China

**Keywords:** chronic stress, tumor microenvironment, glucocorticoid, catecholamine, anticancer treatment

## Abstract

Chronic stress is common among cancer patients due to the psychological, operative, or pharmaceutical stressors at the time of diagnosis or during the treatment of cancers. The continuous activations of the hypothalamic-pituitary-adrenal (HPA) axis and the sympathetic nervous system (SNS), as results of chronic stress, have been demonstrated to take part in several cancer-promoting processes, such as tumorigenesis, progression, metastasis, and multi-drug resistance, by altering the tumor microenvironment (TME). Stressed TME is generally characterized by the increased proportion of cancer-promoting cells and cytokines, the reduction and malfunction of immune-supportive cells and cytokines, augmented angiogenesis, enhanced epithelial-mesenchymal transition, and damaged extracellular matrix. For the negative effects that these alterations can cause in terms of the efficacies of anti-cancer treatments and prognosis of patients, supplementary pharmacological or psychotherapeutic strategies targeting HPA, SNS, or psychological stress may be effective in improving the prognosis of cancer patients. Here, we review the characteristics and mechanisms of TME alterations under chronic stress, their influences on anti-cancer therapies, and accessory interventions and therapies for stressed cancer patients.

## Introduction

Chronic stress, which is associated with the constant activation of the hypothalamic-pituitary-adrenal (HPA) axis and the sympathetic nervous system (SNS) and release of stress hormones including catecholamines and glucocorticoids, occurs frequently in cancer patients during cancer diagnosis and treatment (Gil et al., 2012). The catecholamines and glucocorticoids then activate the adrenergic receptors and glucocorticoid receptors, which belong to the G protein-coupled receptor (GPCR) family and nuclear receptor family respectively, to activate several signaling pathways or alter the transcriptions directly. Unlike the transient secretion of stress hormones in acute stress, lasting elevations of catecholamines and glucocorticoids not only cause mental diseases such as anxiety disorder and depression, but also takes part in the tumorigenesis, progression, metastasis, and drug resistance of various cancers (Reiche et al., 2004). A meta-analysis suggested that stress-related psychosocial factors are associated with higher cancer incidence in initially healthy populations, poorer survival in patients with diagnosed cancer, and higher cancer-related mortality (Chida et al., 2008). Thus, chronic stress is a noteworthy issue in terms of anti-cancer treatments.

Tumor microenvironment (TME), consisting of tumor cells, tumor stromal cells, and non-cellular components, is largely involved in the formation, maintenance, and multidrug resistance (MDR) of cancers (Baghban et al., 2020). Initially, the pro-cancer effects of persistent activations of the HPA axis and SNS under chronic stress are thought to depend mostly on their regulations on systematic immune functions (Sloan et al., 2007; Silverman and Sternberg, 2012). Nowadays, extensive studies have revealed that chronic stress is also responsible for altering the TME, including the tumor cells, cancer stromal cells, and extracellular matrix (ECM), thus participants in cancer-promoting processes.

This review focuses on the consequences of chronic stress on TME and summarizes the characteristics and mechanisms of TME alterations under chronic stress, based on which we emphasize the negative effects of chronic stress on anti-tumor therapies and the implications for formulating well-rounded anti-cancer strategies.

## Features of TME Under Chronic Stress

The TME of patients with chronic stress is distinct from the TME of patients without it, manifested in the differences in the types, statuses, and quantities of immune cells, the class and amounts of cytokines, augmented angiogenesis, enhanced epithelial-mesenchymal transition (EMT), and damaged ECM.

### Immune Cells

**FIGURE 1:**
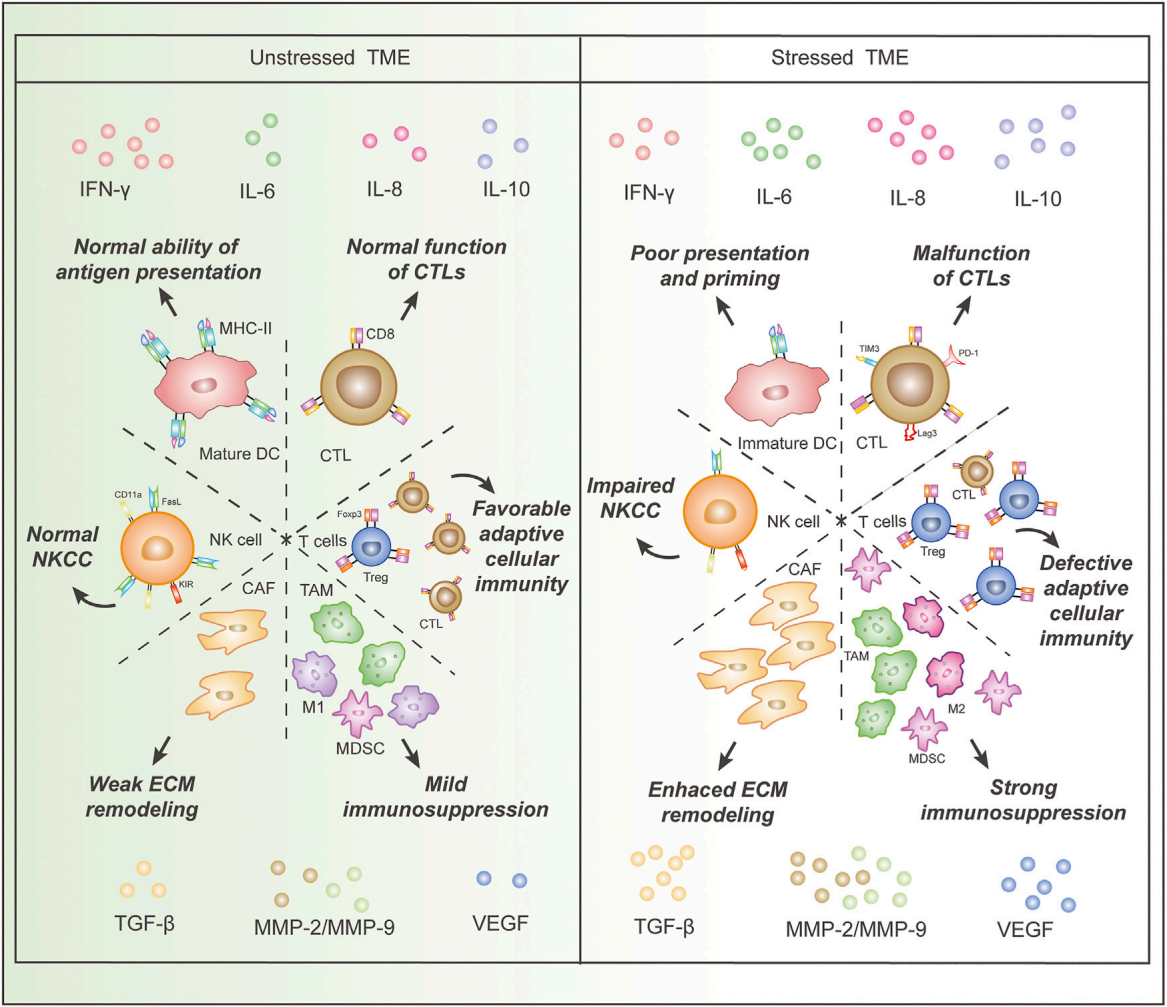
Comparison of immune cells and cytokines in the stressed and unstressed tumor microenvironment (TME). Stressed TME is characterized by (I), decreased proportion and dysfunction of immune-supportive cells, including DCs and CTLs, as well as an increased proportion of cancer-promoting cells, such as MDSCs, Treg cells, CAFs, TAMs, and M2 macrophages; (II), increased concentrations of cytokines that impair anti-cancer immunity and induce angiogenesis, epithelial-mesenchymal transition, and extracellular matrix damage, as well as a decreased concentration of IFN-γ. Abbreviations: IFN, interferon; MMP, matrix metalloproteinase; IL, interleukin; TGF, transforming growth factor; VEGF, vascular endothelial growth factor; DC, dendritic cell; CTL, cytotoxic T lymphocyte, CAF, cancer-associated fibroblast; Treg, regulatory T cell; MDSC, myeloid-derived suppressor cell; TAM, tumor-associated macrophage;NK cell, natural killer cell; FasL, Fas ligand; NKCC, NK cell cytotoxicity; ECM, extracellular matrix.

Generally, the effects of chronic stress on Immune cells in TME are embodied in decreased numbers or functions of immune-supportive cells and increased amounts of exhausted immune cells and immunosuppressive cells (Figure 1).

Dendritic cells (DCs) are essential in tumor antigen presentation and the initiation of cancer adaptive immunity (Gardner and Ruffell, 2016). Nonetheless, Chronic stress or exposure to glucocorticoids disabled immature DCs to undergo full maturation and prime Th1 cells and CD8^+^ T cells efficiently in a rodent model with melanoma, yet the functions of mature DCs were unaffected (Matyszak et al., 2000; Sommershof et al., 2017).

T lymphocytes serve as the main force in cancer adaptive immunity, yet chronic stress leads to a reduction and dysfunction of immune-supportive T cells along with a raise of immunosuppressive T cells (Thommen and Schumacher, 2018). A reduction of cytotoxic T lymphocytes (CTLs) in TME occurred after impaired DC maturation in both healthy mice and the mice with melanoma (Bucsek et al., 2017; Sommershof et al., 2017). Endogenous glucocorticoids inhibited responses of DCs and T cells to type I interferons (IFNs) and IFN-γ, respectively, which compromised the differentiation or activations of these cells in the TME of mice (Yang et al., 2019). Endogenous glucocorticoid signaling led to dysfunctional CD8+ T cells characterized by increased expressions of PD-1, TIM-3, and Lag3 (Acharya et al., 2020). Stress-induced β-AR activation suppressed T-cell receptor (TCR) signaling in a rodent melanoma model and a rodent colon cancer model (Qiao et al., 2021). β2-AR activations in regulatory T (Treg) cells increased their immunosuppressive functions associated with decreased interleukin (IL-2) level and improved differentiation of CD4+ Foxp3- T cells into Foxp3+ Tregs in a rodent model (Guereschi et al., 2013). Stressed mice also had increased suppressive CD25+ cells in tumors of UV-induced squamous cell carcinoma (Saul et al., 2005).

Natural killer (NK) cells, acting through NK cell cytotoxicity (NKCC), represent pivotal cells in tumor innate immunity (DeNardo and Ruffell, 2019). Surgical stress reduced NKCC and NK cell expression of Fas ligand and CD11a in the blood of mice with melanoma or Lewis lung carcinoma (Glasner et al., 2010). Similar diminished NKCC was observed in blood samples from stressed rodent models with leukemia and breast cancer (Ben-Eliyahu et al., 1999). A study on 42 patients with epithelial ovarian cancer revealed that psychological distress was related to lower NK cytotoxicity in TIL (Lutgendorf et al., 2005). Another study revealed impaired NK cell lysis, associated with altered expression of killer immunoglobin-like receptors, in breast cancer patients with high levels of psychological stress (Varker et al., 2007).

Myeloid-derived suppressor cells (MDSCs), presenting in individuals with cancer or chronic stress, play a key role in immune suppression (Gabrilovich, 2017). The operative stress increased the number of immunosuppressive MDSCs in TME (Ma et al., 2019). Similarly, an increase of MDSCs and Treg cells was detected in another stressed male rodent model (Schmidt et al., 2016).

Macrophages are also important components of TME, with which tumors enhance cell proliferation, angiogenesis, and metastasis (DeNardo and Ruffell, 2019). Prostate cancer patients with a higher score of depression revealed higher CD68^+^ tumor-associated macrophage (TAM) infiltration (Cheng et al., 2019), and daily restraint stress increased infiltration of CD68+ macrophages in rodent models of ovarian cancer as well (Colon-Echevarria et al., 2020). Moreover, β2-AR activation promoted macrophages to polarize to immunosuppressive M2 subtype in a rodent breast cancer model (Qin et al., 2015).

In addition, cancer-associated fibroblast (CAF) can regulate TME through cell-cell contact, releasing growth factors, and remodeling the extracellular matrix (Chen and Song, 2019). The activation of α2-ARs boosts the growth and proliferation of fibroblasts, increasing the concentration of growth factors in TME (Bruzzone et al., 2011; Shan et al., 2014).

### Cytokines

Unsurprisingly, the cytokines originating from both tumor cells and stromal cells in stressed TME show cancer-promoting properties (Figure 1). Glucocorticoids reduced the number of IFN-γ-producing cells and the amount of IFN-γ produced in TME of the rodent melanoma model (Matyszak et al., 2000; Sommershof et al., 2017). Increased MDSCs in TME up-regulated transforming growth factor-beta 1 (TGF-β1), vascular endothelial growth factor (VEGF), and Interleukin-10 (IL-10) in rodent breast cancer models (Ma et al., 2019). And glucocorticoid could upregulate the expression of TGF-β receptor type II on ovarian cancer cells and enhance their responsiveness to TGF-β1 (Chen et al., 2010). The activation of β-AR enhanced the secretion of neuropeptide Y (NPY) in a rodent prostate cancer model and subsequently promoted TAM trafficking (Cheng et al., 2019). The level of IL-6 was elevated in the TME of a prostate cancer model due to TAM activation and tumor cell secretion induced by β-AR signaling (Powell et al., 2013; Cheng et al., 2019). Elevations of matrix metalloproteinase (MMP)-9 in TAMs were detected in epithelial ovarian cancer tissue of patients with chronic stress (Lutgendorf et al., 2008). The expressions of VEGF, MMP-2, and MMP-9 were increased in a stressed rodent model of ovarian carcinoma and another stressed rodent model of lung carcinoma (Thaker et al., 2006; Wu et al., 2015), and the same upregulations were detected in nasopharyngeal carcinoma tumor cells treated with norepinephrine (Yang E. V. et al., 2006). Similar upregulations of VEGF and MMP-2 were observed in a rodent oral cancer model (Xie et al., 2015). The upregulated expression of VEGF, IL-8, and IL-6 was also observed in human melanoma tumor cell lines treated with norepinephrine (Yang et al., 2009). Elevated PGE2 secretion was detected in epinephrine-treated *ex vivo* human breast and colon cancer explant and mammary tumors of chronic stress-exposed mice due to activation (Muthuswamy et al., 2017). Additionally, the cytokine analyses in a stressed rodent ovarian cancer model revealed up-regulation of a large scale of cytokines, including platelet-derived growth factor AA (PDGF-AA), epithelial cell-derived neutrophil-activating peptide (ENA-78), angiogenin, VEGF, granulocyte-macrophage colony-stimulating factor (GM-CSF), IL-5, Lipocalin-2, macrophage migration inhibitory factor (MIF), and transferrin receptor (TfR) (Colon-Echevarria et al., 2020).

### Angiogenesis

Overexpression of VEGF and other pro-angiogenic factors like IL-6, TGF-beta, and MMPs, as one of the critical features of stressed TME, leads to enhanced angiogenesis of solid tumors (Kerbel, 2008). This effect was observed in the stressed rodent models of ovarian cancer, oral cancer, and lung cancer, as mentioned above (Thaker et al., 2006; Wu et al., 2015; Xie et al., 2015). Additionally, the expression of VEGFR-2 on endothelial cells was upregulated in the stressed rodent lung cancer model, which also contributes to enhanced angiogenesis (Wu et al., 2015). Moreover, Chronic stress promoted VEGF/FGF2-mediated angiogenesis in a rodent model of breast cancer by down-regulating peroxisome proliferator-activated receptor γ (PPARγ) (Zhou et al., 2020). Enhanced angiogenesis induced by chronic stress and β-adrenergic signaling *via* histone deacetylase-2 (HDAC2)-mediated suppression of thrombospondin-1 was also observed in a stressed model of prostate cancer (Hulsurkar et al., 2017). What’s worse, chaotic and unfunctional vessels induced by intense angiogenesis lead to other problems like acidosis and hypoxia in TME (Neri and Supuran, 2011; Rey et al., 2017).

### Epithelial-Mesenchymal Transition

As TGF-β family signaling is crucial in EMT, chronic stress also promotes EMT because TGF-β1 is markedly upregulated in stressed TME (Lamouille et al., 2014). A high concentration of TGF-β1 induces EMT of tumor cells and promotes tumor metastasis in stressed rodent models with breast cancer (Ma et al., 2019). Norepinephrine induced EMT, reflected in E-cadherin downregulation and vimentin upregulation, *via* β-AR/TGF-β1/p-Smad3/Snail pathway or β-AR/TGF-β1/HIF-1α/Snail pathway in gastric, colonic, and pneumonic cancer cell lines *in vitro* (Shan et al., 2014; Zhang et al., 2016). In addition, Chronic stress downregulates E-cadherin expression and upregulates vimentin expression through the activation of miR-337-3p/STAT3 in a stressed rodent model with breast cancer (Du et al., 2020).

**FIGURE 2:**
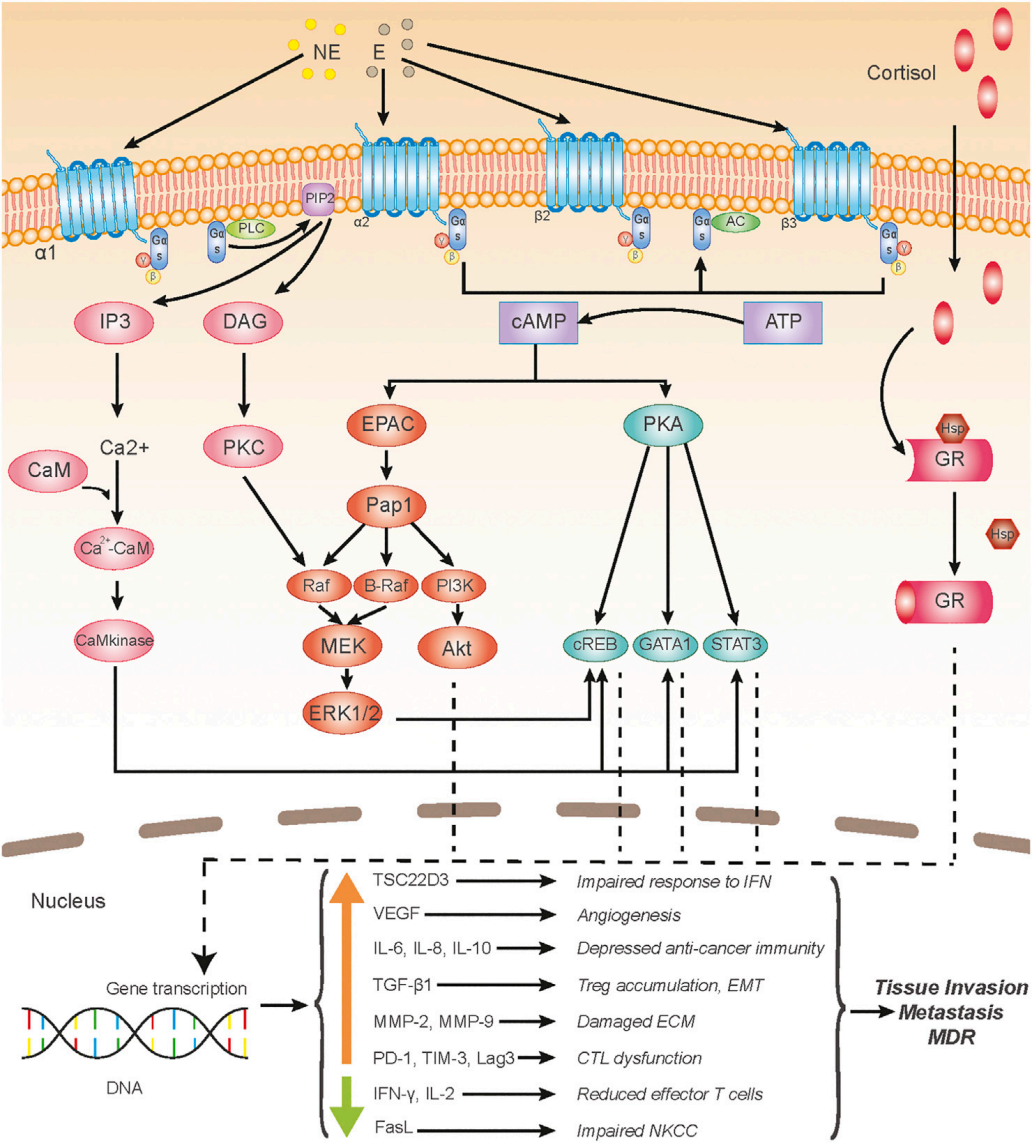
Chronic stress-induced signaling pathways acting upon tumor microenvironment. Adrenergic receptors (ARs), including α-AR, β2-AR, and β3-AR get involved in chronic stress-induced tumor microenvironment (TME) alterations; ARs are G protein-coupled receptors (GPCRs), the binding of AR agonists, such as norepinephrine and epinephrine, to which activates intracellular Gαs protein. Activated Gαs either activates PLC (α1-AR) or AC (α1-AR, β2-AR, and β3-AR), which subsequently induces an intracellular increase of IP3 and DAG, or cAMP, respectively, and then the second messengers initiate activation of several signaling pathways, including the PKA, PKC, EPAC, and Ca^2+^-CaM pathways. Glucocorticoid receptors (GRs), consisting of the glucocorticoid-binding subunit and Hsp 90 protein, belong to the nuclear receptor family and locate intracellularly; after glucocorticoids permeate through the cell membrane and bind to GRs, the Hsp protein depolymerizes from the polymeric substance and the main subunits of GRs translocate into the nucleus and initiate gene transcriptions. Transcriptions of various genes of cytokines or ligands are up-regulated or down-regulated, altogether causing the deterioration of the TME, which leads to a large scale of cancer-promoting effects. Abbreviation: NE, norepinephrine; E, epinephrine; PLC, phospholipase C; PIP2, phosphatidylinositol (4,5) bisphosphate; AC, adenylatecyclase; IP3, inositol triphosphate; DAG, diacylglycerol; CaM, calmodulin; PKC, protein kinase C; ATP, adenosine triphosphate; cAMP, cyclic adenosine monophosphate; PKA, protein kinase A; EPAC, exchange protein activated by adenylyl cyclase; Pap1, production of anthocyanin pigment 1; PI3K, phosphatidylinositol 3-kinase; MEK, mitogen-activated protein kinase kinase (MAPKK); ERK, extracellular regulated protein kinase; CREB, cAMP-responsive element-binding protein; GATA1, GATA Binding Protein 1; STAT3, signal transducer and activator of transcription 3; GR, glucocorticoid receptor; Hsp, heat shock protein; IFN, interferon; NKCC, NK cell cytotoxicity; ECM, extracellular matrix; CTL, cytotoxic T lymphocyte; EMT, epithelial-mesenchymal transition; MDR, multidrug resistance.

Moreover, given that the activation of ARs can induce the activations of protein kinase A (PKA) and protein kinase C (PKC) (Biazi et al., 2018; Durkee et al., 2019; Cole and Sood, 2012) (Figure 2), chronic stress can be associated with EMT *via* PKA and PKC signaling. It is well established that PKC promotes EMT by activating various downstream molecules. PKCα was regarded as a central signaling node for EMT in breast cancer (Tam et al., 2013). PKCθ was reported to induce EMT through TGF-βand NF-κB signaling (Zafar et al., 2014; Zafar et al., 2015), and PKCδ could induce EMT via phosphorylation of Bcl-2 associated athanogene 3 (BAG3) (Li et al., 2013). Moreover, a study showed that PKC-induced EMT was associated with a down-regulation of carbonic anhydrase 12 (CAXII) (Vergara et al., 2020). In contrast, the activation of PKA favored the epithelial type and contributed to the mesenchymal-epithelial transition (MET) of the tumor cells (Nadella et al., 2008; Pattabiraman et al., 2016). Yet, research showed that PKA promoted TGF-βinduced EMT (Yang Y. et al., 2006), and enhanced activity of PKA plays an important role in hypoxia-mediated EMT (Shaikh et al., 2012). However, research on PKA/PKC-induced EMT using stress or stress hormone-treated models is lacking, so the exact roles of PKA and PKC in chronic stress-induced EMT still need further investigations.

Additionally, it is notable that chronic stress is also associated with deteriorations of gut microbiota (Gao et al., 2018), which can facilitate EMT through microbiota-host interactions (Vergara et al., 2019).

### Extracellular Matrix

Elevations of MMPs were present in TME of various cancers (Thaker et al., 2006; Lutgendorf et al., 2008; Wu et al., 2015; Xie et al., 2015), which are likely to cause damages to ECM and promote cancer metastasis (Najafi et al., 2019). Additionally, glucocorticoids can downregulate the synthesis of tenascin-C, a vital protein in the extracellular matrix, in a rodent Wilms’ tumor model, even though local stimulatory growth factors are present (Talts et al., 1995).

### Metabolism

Chronic stress can cause molecular and functional recalibrations of mitochondria and metabolic disorders in immune cells (Picard and McEwen, 2018; Fan et al., 2019), which can alter the metabolic features in TME. Also, hostile TME with limited oxygen and nutrients can lead to metabolic reprogramming of local T cells and impair their functions (Pearce et al., 2013). Chronic stress-induced up-regulation of epinephrine could activate lactate dehydrogenase A (LDHA) to generate lactate and promote breast cancer stem-like properties in a rodent model (Cui et al., 2019). Besides, β-AR activation depressed endothelial oxidative phosphorylation and turned on the angiogenic switch for tumor progression in a rodent prostate cancer model (Zahalka et al., 2017). Additionally, the activations of PKC and PKA can lead to unfavorable metabolic alterations and fuel cancer progression (Aggarwal et al., 2019; Liu et al., 2019; Vergara et al., 2020).

## Mechanisms for TME Alterations Under Chronic Stress

The TME alterations under chronic stress are mainly derived from activated AR signaling and glucocorticoid signaling. Under chronic stress, SNS is constantly activated, resulting in a high concentration of catecholamine in solid tumor tissues, which drives from both circulating blood and local sympathetic neurons (Reiche et al., 2004). Additionally, endogenous glucocorticoids, deriving from the adrenal cortex, of which the concentration in the blood increases under chronic stress *via* the HPA axis, easily diffuse across the membrane of various cells in TME (Kadmiel and Cidlowski, 2013). One thing to point out here is that the enhanced β-adrenergic signaling and glucocorticoid signaling in TME can be induced by not only chronic stress, but also TME hypoxia (Chiarugi and Filippi, 2015).

α-AR signaling is partly responsible for TME alterations. There are two subtypes of α-ARs, including α1-AR and α2-AR, both of which belong to the GPCR family (Taylor and Cassagnol, 2021). The activation of α1-AR leads to the increase of intracellular calcium concentration *via* the PLC-IP3/DAG pathway, while the activation of α2-AR results in the inhibition of adenylyl cyclase, which decreases the concentration of cytoplasmic calcium and cAMP (Biazi et al., 2018; Durkee et al., 2019) (Figure 2).

β-ARs are expressed on the membranes of various tumor cells and tumor-related cells, such as immune cells, fibroblast, and epithelial cells, and epithelial cells, and two of the three subtypes of β-ARs, including β2-AR and β3-AR, take part in tumorous β-signaling (Daly and McGrath, 2011; Calvani et al., 2020). The binding of catecholamine, like epinephrine and norepinephrine, to β-ARs, contributes to the activation of guanylate cyclase, leading to transient cyclic adenosine monophosphate (cAMP) flux, which subsequently activates protein kinase A (PKA) and guanine nucleotide exchange protein activated by adenylyl cyclase (EPAC) (Cole and Sood, 2012). The two latter proteins activate a variety of intracellular pathways which switch on or off the transcriptions of genes, associating with inflammation, angiogenesis, tissue invasion, distant metastasis (Thaker et al., 2006; Cole and Sood, 2012; Duan et al., 2019) (Figure 2).

Glucocorticoids can be produced by the adrenal cortex and translocated to the tumor, or produced locally by TAMs (Acharya et al., 2020). Glucocorticoid receptors (GRs), located intracellularly, once activated by glucocorticoid, get involved in the formation of a complex and translocate to nuclei, which induce transcriptionally activation or suppression of gene expressions *via* direct interactions with DNA (Timmermans et al., 2019). For instance, TSC22D3 is upregulated in response to stress by glucocorticoid signaling, which blocks the response of DC to type I IFN and IFN-γ^+^ T cell activation (Yang et al., 2019) (Figure 2).

## Chronic Stress Influents Anti-Cancer Therapy

It has been widely accepted that the TME profile plays a dominant role in determining the efficacies of anti-cancer therapies (Roma-Rodrigues et al., 2019). Not surprisingly, the TME alterations under chronic stress have negative impacts on the efficacies of cancer treatments, including chemotherapy, immunotherapy, and targeted therapy.

### Chemotherapy

Chronic stress-induced endogenous glucocorticoids have unfavorable effects on the therapeutic response to chemotherapy. Dexamethasone increased the adhesion to ECM and the resistance to cisplatin and paclitaxel in two human ovarian cancer cells (Chen et al., 2010). The therapeutic of oxaliplatin (OXA)-based chemotherapy effect was largely compromised in social-defeat (SD)-conditioned mice (Yang et al., 2019). And a high expression of the glucocorticoid receptors (GR) was correlated with shorter metastasis-free survival in triple-negative breast cancer (TNBC) patients undergoing chemotherapy (Chen et al., 2015).

Endogenous catecholamines, such as norepinephrine and epinephrine, also interfere with chemotherapy. Catecholamines reduced p53 protein concentrations in cancer cells and increased the genetic instability of these cells, which significantly inhibited paclitaxel-induced and cisplatin-induced apoptosis in ovarian cancer cells (Hara et al., 2011; Kang et al., 2016). Yet, reduction of stress-related signaling potentiated the effect of chemotherapy in cancer patients has also been demonstrated (Mravec et al., 2020).

### Immunotherapy

Chronic-stress induced reductions of CD8+ T cells and CTLs result in a cancer vaccine failure in a rodent melanoma model (Sommershof et al., 2017). Dysfunction of CD8^+^ TILs induced by endogenous glucocorticoid signaling is associated with failure to respond to checkpoint blockade in both preclinical models and melanoma patients (Acharya et al., 2020). Increased infiltration of regulatory T-cells, decreased amount of CD8+ lymphocytes in tumor sites were observed in bladder-tumor-bearing mice treated with anti-PD-L1 under chronic stress. Therefore, chronic psychological stress could weaken the potency of anti-PD-L1 immunotherapy (Zhou et al., 2021).

A glucocorticoid-inducible molecule, TSC22D3 plays an important role in stress-induced immunosuppression as well as perturb responses to prophylactic tumor vaccination and PD-1-targeted immunotherapy (Yang et al., 2019). In addition, psychological stress down-regulated the expression of interleukin-2 (IL-2) receptor in peripheral blood leukocytes, affects the therapeutic efficacy of IL-2 immunotherapy in renal cell cancer patients (Zhang et al., 2020).

### Molecule-Targeted Therapy

Since chronic stress increases VEGF secretion in TME, it can impair the efficacies of anti-angiogenic agents. By upregulating the VEGF expression *via* the β-AR-cAMP-PKA signaling pathway, chronic stress and exogenous norepinephrine markedly weakened the efficacy of sunitinib in rodent models of colorectal cancer and melanoma respectively (Deng et al., 2014; Liu et al., 2015).

Chronic stress-induced stress hormone norepinephrine (NE) promotes afatinib resistance by upregulating Cx32 expression which could decrease the degradation of EGFR-TKI resistance-associated proteins (MET, IGF-1R) and increase their transcription levels (Xie et al., 2019). β2-AR activation on non-small cell lung cancer (NSCLC) cell induces epidermal growth factor receptor (EGFR) tyrosine kinase inhibitor (TKI) resistance by inactivating liver kinase B1 (LKB1), elevating IL-6 expression, and MAPK pathway in a rodent model (Nilsson et al., 2017). Indeed, in treatment-naive patients with advanced lung adenocarcinoma receiving first-line EGFR-TKIs, prior b-blocker use was associated with a longer time-to-discontinuation (TTD) and overall survival (OS) (Chang et al., 2020).

## Anti-Cancer Treatments and Interventions Targeting Chronic Stress

### β-Blocker

β-blockers, such as propranolol and metoprolol, can block the interactions between catecholamine and β-AR, which inhibits the subsequent cancer-promoting effects induced by β-AR signaling as mentioned above (Fumagalli et al., 2020). Blocking β-AR interrupts the differentiation of exhausted T progenitors and decreases the number of exhausted T cells in TME (Qiao et al., 2021). Propranolol reduced MDSC accumulation in the TME of thermal-stressed mice treatments and controlled tumor growth (MacDonald et al., 2021). Blocking β-AR also increases glycolysis and oxidative phosphorylation in tumor-infiltrating lymphocytes (TIL), which leads to increased CD28 expression and enhanced anti-tumor functions (Qiao et al., 2021). Propranolol can enhance the sensitivity of gastric cancer cells to radiation *in vitro* by inhibiting NF-κB-VEGF/EGFR/COX-2 pathway (Liao et al., 2010).

Combined administration of propranolol and etodolac, a cyclooxygenase-2 inhibitor, improved the survival rate of mice with melanoma or Lewis lung carcinoma (Glasner et al., 2010). A prospective pilot study showed that the combination of propranolol with chemotherapy improved the quality of life (QOL) of patients with epithelial ovarian cancer (Ramondetta et al., 2019).

The combination of propranolol with targeted therapy may improve the efficacy. An exploratory analysis of the LUX-Lung3 study revealed a significant PFS prolongation of NSCLC patients taking β-blockers with EGFR-TKI afatinib compared with those taking afatinib alone (median 11.1 vs. 6.9 months, *p* = 0.0001), indicating there is a synergic effect combining β-blockers with anti-EGFR therapy (Nilsson et al., 2017).

As the favorable efficacy of immunotherapy is based on the premise of an immune-supportive TME, β-blockers may be ideal companions for immunotherapy owing to their capacity of inhibiting β-AR-induced TME deterioration. The increased density of CTLs and decreased expression of PD-1 induced by propranolol enhanced the efficacy of anti-PD-1 agents in a rodent model. Propranolol strongly improved the efficacy of an anti-tumor STxBE7 vaccine by enhancing the frequency of CTLs in a rodent model (Daher et al., 2019). Propranolol increased the concentration of IL-12, IL-17, 1L-2, and IFN-γ in the breast tumor of mice and assisted with a tumor lysate vaccine (Ashrafi et al., 2017). A phase I study showed promising safety and activity of combining propranolol and pembrolizumab in the first-line treatment of metastatic melanoma (Gandhi et al., 2021). A meta-analysis revealed that β-blockers significantly improved DFS (HR 0.03, 95% CI 0.01–0.17) and OS (HR 0.04, 95% CI 0.00–0.38) in melanoma patients, but the beneficial effect is quite tumor-specific (Yap et al., 2018).

### α-Blocker

α-blockers can also function as anti-cancer agents. Quinazoline α-1 blockers, such as prazosin, doxazosin, and terazosin, have shown promising anti-cancer effects in various types of cancer, and benefit chemotherapy, radiotherapy, and anti-EGFR therapy (Ashrafi et al., 2017). The VEGF-induced angiogenesis is inhibited by an α-blocker, doxazosin, in human umbilical vein endothelial cells (Keledjian et al., 2005). Another selective α1-blocker, naftopidil, presents with anti-proliferative and cytotoxic effects on prostate cancer as well as several other cancer types *in vitro*, *ex vivo*, and *in vivo* (Florent et al., 2020).

### Stress-Reducing Interventions

Moreover, interventions targeting directly on physical or psychological stressors may ameliorate the TME and benefit anti-cancer treatments as well. The activation of the brain reward system decreased SNS activity and β-adrenergic signaling, which led to less immunosuppressive MDSCs in a murine model (Ben-Shaanan et al., 2018). Thermal treatments, including weekly whole-body hyperthermia and housing mice at their thermoneutral or sub-thermoneutral temperature, also decreased MDSC accumulation and tumor growth of mice (MacDonald et al., 2021). A similar enhancement of immune checkpoint inhibitor efficacy was observed in a rodent model with physiologically reduced stress (Bucsek et al., 2017). Mice housed in an enriched environment displayed enhanced NK-cell activity and increased infiltration of NK cells into TME (Song et al., 2017). Stress-reducing approaches, such as yoga, mindfulness, and cognitive behavioral therapy, have shown broad clinical benefits of increasing proportions of anti-tumor immune cells and cytokines in several studies, yet the results of these studies were limited with small sample sizes and short follow-ups (Antoni and Dhabhar, 2019).

### Others

Additionally, a variety of other drugs may also reverse the unfavorable TME alterations in terms of chronic stress. Zoledronic acid, an anti-cancer adjuvant drug, is proficient in abrogating stress-induced macrophage infiltration, and PDGF-AA expression in a rodent ovarian cancer model (Colon-Echevarria et al., 2020). Antidepressants, such as fluoxetine and sertraline, can also alleviate chronic stress and have the potential in associating with cancer treatments, which still need further clinical confirmation (Di Rosso et al., 2018).

Some other strategies targeting chronic stress-inducible inflammatory signaling have shown promising efficacies in clinical practice. Bevacizumab, an anti-VEGF agent, is now applicable for a wide range of solid tumors and has shown favorable efficacies combined with chemotherapy, immune checkpoint inhibitors, anti-EGFR therapy, and PARP inhibitors (Garcia et al., 2020). Anti-IL-6 or Anti-IL-6 receptor agents, such as tocilizumab and siltuximab, have not shown satisfactory efficacies in cancers (Rossi et al., 2015), yet tocilizumab has been widely used to treat cytokine release syndrome induced by CAR T-cell toxicity (Brudno and Kochenderfer, 2019).

## Discussion

In summary, the TME under chronic stress is differentiated from others by increased numbers and enhanced functions of immunosuppressive cells, decreased amounts and impaired functions of immunosupportive cells, associated with corresponding changes in cytokines, which results in intense angiogenesis, boosted tumor cell proliferation and enhanced EMT inside of the TME. The over-secretion of glucocorticoid and catecholamines deriving from persistent activations of the HPA axis and SNS mostly contribute to the TME alterations under chronic stress. These alterations can reduce the efficacies of anti-tumor therapies, like chemotherapy, immunotherapy, and targeted therapy. Drugs, such as α-blockers, β-blockers, antidepressants, and interventions, like meditation and mindfulness, may cut down the negative effects of chronic stress, which should draw the attention of clinical oncologists in adopting treatment plans for patients with chronic stress.

Still, recent studies for the interactions of chronic stress and TME have limitations, such as absences of evaluation of animal stress levels within the group and assessments for stress levels before investigations in animal models (Hylander et al., 2019). Therefore, researchers should value the importance of stress quantification in research, and approaches, such as detections of serum glucocorticoids and catecholamines before further procedures, should be taken to control potential bias.
