# Corrigendum: Chronic Stress: Impacts on Tumor Microenvironment and Implications for Anti-Cancer Treatments

**DOI:** 10.3389/fcell.2022.865043

**Published:** 2022-03-04

**Authors:** Wentao Tian, Yi Liu, Chenghui Cao, Yue Zeng, Yue Pan, Xiaohan Liu, Yurong Peng, Fang Wu

**Affiliations:** ^1^ Department of Oncology, The Second Xiangya Hospital, Central South University, Changsha, China; ^2^ Xiangya School of Medicine, Central South University, Changsha, China; ^3^ Xiangya School of Public Health, Central South University, Changsha, China; ^4^ Hunan Cancer Mega-Data Intelligent Application and Engineering Research Centre, Changsha, China; ^5^ Hunan Key Laboratory of Tumor Models and Individualized Medicine, The Second Xiangya Hospital, Central South University, Changsha, China; ^6^ Hunan Key Laboratory of Early Diagnosis and Precision Therapy in Lung Cancer, The Second Xiangya Hospital, Central South University, Changsha, China

**Keywords:** chronic stress, tumor microenvironment, glucocorticoid, catecholamine, anticancer treatment

In the original article, there were some mistakes in Figure 1 as published. 1) In the right part of Figure 1, “PD-L1” should be “PD-1”. 2) In the right part of Figure 1, “ITM-3” should be “TIM-3”. 3) In the right part of Figure 1, “dedective” should be “defective”. The corrected Figure 1 appears below.

**FIGURE 1:**
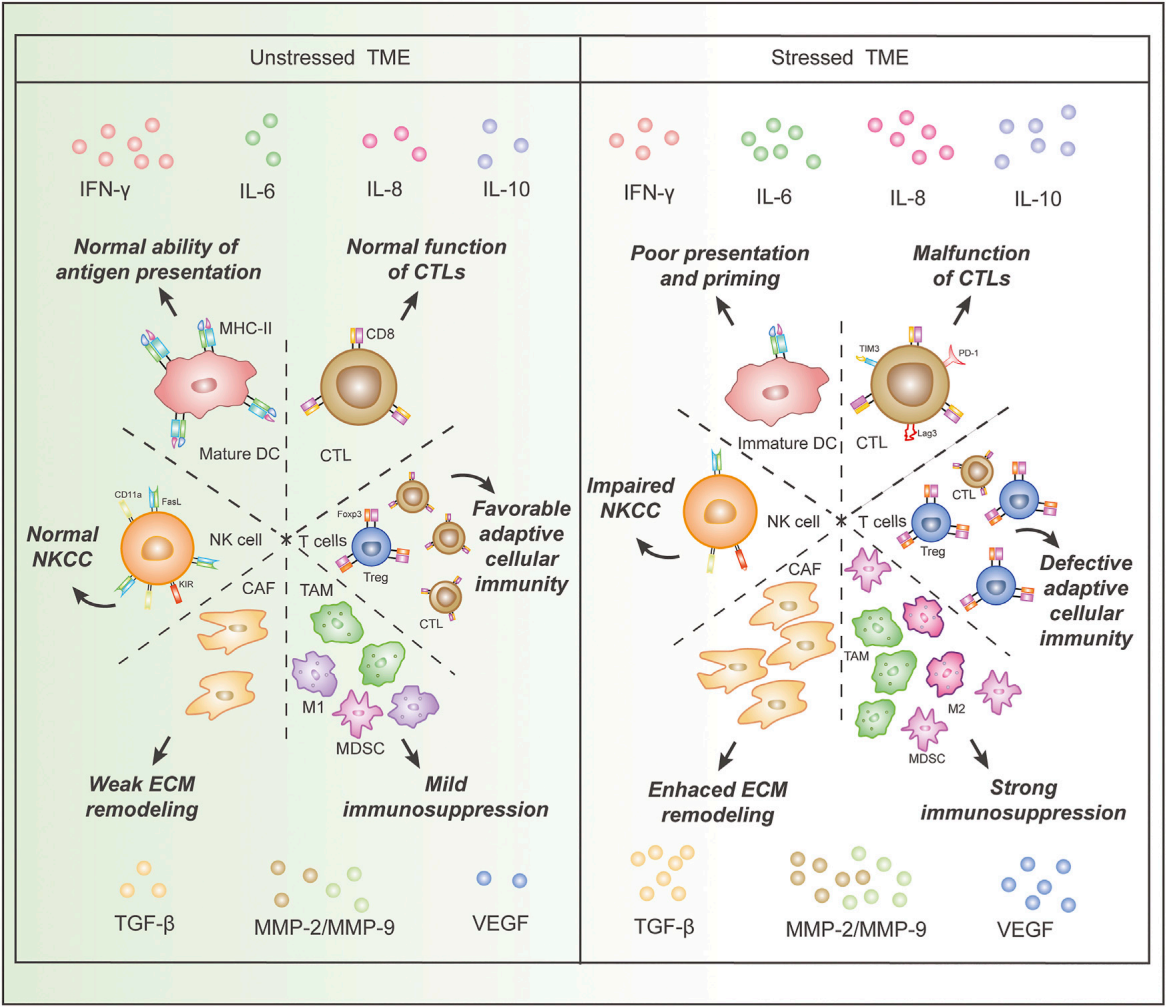
Comparison of immune cells and cytokines in the stressed and unstressed tumor microenvironment (TME). Stressed TME is characterized by (I), decreased proportion and dysfunction of immune-supportive cells, including DCs and CTLs, as well as an increased proportion of cancer-promoting cells, such as MDSCs, Treg cells, CAFs, TAMs, and M2 macrophages; (II), increased concentrations of cytokines that impair anti-cancer immunity and induce angiogenesis, epithelial-mesenchymal transition, and extracellular matrix damage, as well as a decreased concentration of IFN-γ. Abbreviations: IFN, interferon; MMP, matrix metalloproteinase; IL, interleukin; TGF, transforming growth factor; VEGF, vascular endothelial growth factor; DC, dendritic cell; CTL, cytotoxic T lymphocyte, CAF, cancer-associated fibroblast; Treg, regulatory T cell; MDSC, myeloid-derived suppressor cell; TAM, tumor-associated macrophage;NK cell, natural killer cell; FasL, Fas ligand; NKCC, NK cell cytotoxicity; ECM, extracellular matrix.

In the original article, there was an error in the section, **Anti-Cancer Treatments and Interventions Targeting Chronic Stress, β-Blocker**. “The combination of propranolol and immune checkpoint inhibitors may improve the efficacy of immunotherapy.” should be “The combination of propranolol with targeted therapy may improve the efficacy.”, as the following part talks about targeted therapy.

A correction has been made to **Anti-Cancer Treatments and Interventions Targeting Chronic Stress, β-Blocker**, Paragraph 3:

“The combination of propranolol with targeted therapy may improve the efficacy. An exploratory analysis of the LUX-Lung3 study revealed a significant PFS prolongation of NSCLC patients taking β-blockers with EGFR-TKI afatinib compared with those taking afatinib alone (median 11.1 vs. 6.9 months, *p* = 0.0001), indicating there is a synergic effect combining β-blockers with anti-EGFR therapy (Nilsson et al., 2017)”.

The authors apologize for this error and state that this does not change the scientific conclusions of the article in any way. The original article has been updated.

